# Digital Economy and Health: A Case Study of a Leading Enterprise's Value Mining Mode in the Global Big Health Market

**DOI:** 10.3389/fpubh.2022.904186

**Published:** 2022-08-17

**Authors:** Jialin Guan, Huijuan Xu, Yunfeng Wang, Yaoguang Ma, Yu Wang, Rui Gao, Kexuan Yu

**Affiliations:** School of International Economics and Trade, Jilin University of Finance and Economics, Changchun, China

**Keywords:** Keeson, digital economy, global big health market, Bayesian model, health and elderly care

## Abstract

Coronavirus disease 2019 (COVID-19) swept across the world and posed a serious threat to human health. Health and elderly care enterprises are committed to continuously improving people's health. With the rapid development of the digital economy, many enterprises have established digital product-service ecosystems after combining “Internet +,” big data, cloud computing, and the big health industry. This paper uses the case study method to analyze the overseas market value mining mode of health and elderly care enterprises through in-depth research on leading health and elderly care enterprises. This study explores the value mining mode of the leading enterprise's global big health market using a cluster analysis and Bayesian model with the support of data on geographical characteristics, users' sleep habits, and national big health. This paper theoretically summarizes the successful cases of health and elderly care enterprises through digital transformation, which provides a useful reference for the intelligent transformation of the health and elderly care industry.

## Introduction

With the deep integration of “Internet +,” big data, and the real economy, the digital economy has entered a new era. In the digital economy era, innovation and upgrading in manufacturing have been promoted by the revolution of cloud computing, the internet, and information technology. The innovation of the manufacturing industry depends not only on product upgrading but also on the breakthroughs of industrial boundaries. This means that the vitality of the industry can be stimulated by boundless thinking to enhance the competitiveness of Chinese manufacturing in the global value chain. Boundless innovation refers to the integration and innovation of enterprises across borders. Boundless innovation thinking is about an enterprise that promotes the innovation of products, services, and business modes based on a comprehensive user experience to meet the increasingly diverse needs of consumers. This is especially true when an enterprise is breaking down industrial barriers and connecting the upstream and downstream segments of the industrial chain with Internet of Things, big data, and cloud computing.

As coronavirus disease 2019 (COVID-19) has spread globally since February 2020, quarantine is the best way to avoid being infected. Many countries imposed a major shutdown of non-essential business and restricted all non-essential outdoor movements. Quarantines around the world have significantly increased the amount of time people spend at home. In this context, people pay more attention to health and smart home products.

We have chosen Keeson Technology Corporation Limited (hereinafter referred to as Keeson) as the subject of this study. Keeson is the official supplier of smart beds for the Beijing 2022 Olympic and Paralympic Winter Games. It is also one of the world's largest exporters of smart beds and a leader of China's health and elderly care enterprise. It is mainly engaged in the research and development (R&D), design, production, and sales of smart beds, and other accessories. It is a multinational enterprise committed to integrating products and smart technologies with big health data. Its products are exported to North America, Asia, Europe, Oceania, and other regions. Under the background of the digital economy, this study aims to analyze how Keeson creates core competitiveness with the boundless innovation mode. Using a cluster analysis and a Bayesian model, this study selects the best overseas market for Keeson.

The marginal contribution of this study is mainly reflected in two aspects. First, this study takes the leading health and elderly care enterprises as the research subject to explore the theoretical mechanism of how health and elderly care enterprises build a digital ecosystem of products and services through boundless innovation; second, this study refines the overseas value mining mode of health and elderly care enterprises, and puts forward suggestions on the overseas value mining paths, which provide a valuable reference for other health and elderly care enterprises.

This study is carried out from the following aspects. Section Literature Review deals with the literature review, which summarizes the relevant literature on the innovative business modes of the health and elderly care industry and the strategic export market layout in the context of the digital economy. Section Theoretical Analysis discusses the theoretical analysis, which summarizes the theoretical logic of the boundless mode innovation of health and elderly care enterprises from the service leading logic, smile curve and value chain theory, long tail theory, and springboard theory. Section Empirical Analysis deals with the empirical analysis, which empirically tests the overseas value mining mode of leading enterprises through a cluster analysis and a Bayesian model. Section Value Mining Strategy for the Global Big Health Market deals with the suggestions on the overseas market value mining mode of health and elderly care enterprises. The last part discusses the implications and limitations of refining the boundless innovation mode of health and elderly care enterprises and of providing a reference for other enterprises.

## Literature Review

### The Digital Economy and the Health and Elderly Care Industry

The digital economy is an effective interaction between new economies, new businesses, and new technologies (1). The digital economy is the integration of computer and communication technology on the internet, leading to the exchange of information and technology (2). Scholars have carried out research related to the economic effects of the digital economy. The results show that the digital economy has a positive impact on income growth (3), production efficiency improvement (4), industrial structure adjustment (5), business model innovation (6), and urban development (7). Meanwhile, government plays an important role in promoting the healthy development of the digital economy (8).

In recent years, the smart health and elderly care industry has gradually become a new trend to promote innovation and development (9). Intelligent innovation includes intelligent product R&D, intelligent production and manufacturing, intelligent marketing management, intelligent service, etc. (10). Intelligent manufacturing based on high-technology services, such as “Internet +” and artificial intelligence, is the key for traditional Chinese health and elderly care enterprises to improve their independent innovation ability (11).

As a new driving force and new element of high-quality economic development in the new era, the digital economy has gradually become a key factor in solving the development of the health and elderly care industry. By using big data technologies, data from the health and elderly care industry can be intelligently collected, integrated, analyzed, and shared (12). The integration of the digital economy in the health and elderly care industry improves service quality (13), improves production efficiency, reduces production costs, and increases production capacity (14).

### Innovation and the Development of Traditional Manufacturing Enterprises

Accelerating the exploration of innovation by enterprises is considered as a threshold for high-quality development. At present, scholars at home and abroad are actively seeking ways for traditional manufacturing enterprises to break through the smiling curve dilemma.

It is suggested that the health and elderly care industry can achieve high-quality development by upgrading the industrial chain. From this point of view, enterprises need to improve their R&D capacity, open up the left end of the smiling curve or reshape their own brand, and extend the right end of the curve based on production and manufacturing (15, 16).

Servitization is also believed to be an important path for manufacturing innovation and development (17). Taking the value chain as the subject of research, Jian and Wu proposed the following four paths for manufacturing industry servitization: upstream integration, downstream integration, upstream–downstream integration, and demanufacturing (18). Luo et al. proposed a service-oriented manufacturing innovation pathway, which is divided into four levels, namely, the after-sales service level, differentiated service level, value-added service level, and experience service level (19).

### A Strategic Layout of the Enterprise Export Market

Diversification of export markets not only leads to an increase in exports, but also reduces overall risk, which is one of the manifestations of enterprise expansion. However, enterprises do not export to all markets. In fact, there are a large number of zero points in the trade matrix. In other words, enterprises export selectively to certain markets (20). Therefore, many scholars have started to focus on the selection of export markets rather than on the export identities of enterprises.

One stream of research is to select markets based on export experience. Scholars have generally suggested that enterprises have a path dependence in the choice of new export markets (21–24). The main point of this view is that enterprises are more inclined to enter new markets similar to existing export markets. Existing and potential markets can be culturally recognized or geographically close (25). Even the existing market border can become a new market (26). Based on these connections, enterprises can take the export experience of the existing market as a springboard to new markets (27).

Some scholars choose markets *via* small-scale export testing. Due to the uncertainty of export markets, enterprises can learn about the demand quantity (28) and consumer preferences in the target market through an export test. Then, enterprises judge the matching degree between their products and the needs of the target market and whether or not enterprises can make a profit (29). Sometimes, these tests can be successful, leading to a rapid growth for of the enterprises' exports. However, the tests sometimes fail, causing enterprises to withdraw their products from foreign markets (30).

Some scholars have proposed the selection methods for export markets from other perspectives. Chaney and Eaton et al. have suggested that enterprises will enter all markets where zero profit productivity is lower than their own productivity, rather than exporting to markets with thresholds higher than their own productivity (31, 32). Ma and Lv use enterprise productivity to select large-scale export markets (33). They hold the idea that enterprises with high productivity levels can enter the markets of developed countries, while enterprises with lower productivity levels can export products to the markets of developing countries. He suggests that increased proportion of the long-tail market makes it easier for enterprises to conduct foreign trade marketing (34). Proper application of the long-tail market can create more value for foreign trade enterprises. Cai et al. establish an export market portfolio model to determine the export share of each market in the optimal geographic export direction to select the best export market for Chinese goods worldwide (35).

In general, export experience and small-scale export tests are both feasible methods to select target markets through empirical tests, but both have certain limitations. The former selects a market based mainly on geographical locations with high concentrations, which does not meet the strategic needs of enterprises' global layout. The latter involves multiple high-cost tests that are time-consuming and have low efficiency. In addition, although some scholars choose export markets according to the enterprise's own ability, most of them take productivity as the selection criterion and neglect some irreplaceable capabilities, such as small-scale technology to meet long-tail market demand and customized after-sales service. In short, existing research has not yet reached a consensus on a set of effective methods for enterprises to choose export markets, especially for health and elderly care enterprises in transition and upgrading.

## Theoretical Analysis

Smart health and elderly care is an inevitable trend stemming from the penetration of the network and control technology in the health and elderly care industry. Guided by “Internet +,” the invisibility of consumer consumption and purchases further promotes the boundless development of the health and elderly care industry and the independent innovation by enterprises. On the left side of the smiling curve, guided by the long-tail theory, Keeson focuses on self-innovation and independent R&D, aiming to meet the needs of 98% of consumers and varying sub-customary consumption as much as possible. In the middle of the smiling curve, Keeson increases the added value of its products through intelligent manufacturing. In this way, Keeson's products are more competitive, thus attracting more free potential customers. At the right end of the smiling curve, Keeson opens up its boundaries and obtains feedback from consumers to further update its products and services. Through co-creation with consumers, Keeson forms a unique digital ecosystem and gradually upgrades itself. Based on a relatively complete digital ecosystem, Keeson will gradually achieve its goals of internationalization and high-quality development of foreign trade.

### Service-Dominant Logic

With the continuous improvement of the quality of life and living standards, consumers' concept of consumption has gradually changed from buying daily necessities to the simultaneous pursuit of products and services. The long-running dispute between goods and services has gradually escalated, and scholars have engaged in a fierce debate over a commodity-dominated logic and service dominance. The commodity-dominant logic was formed against the background of the industrial revolution when job specialization was deeply rooted in people's minds. However, in today's era, the boundaries between goods and services have become increasingly blurred. Service has become one of the essential elements that manufacturing enterprises must provide. Keeson constructs its future blueprints through the servitization of manufacturing, and enhance their own competitiveness through the process of co-creation with customers. Vargo and Lusch suggested following the new service-dominated logic to discuss the relationship between goods and services (36). Instead of distinguishing between primary and secondary or the advantages and disadvantages of the two, goods and services should be unified under service and the basic issues of market transaction and value creation should be rethought. Service is not only an extension of the enterprise's own value, but also a form of dialogue between enterprises and consumers. After a long period of modification and improvement of the initial theoretical framework, a mature system of service-dominant logic theory has been gradually formed (37). A total of 10 propositions put forward by the service-dominant logic indicate that enterprises should take service as the basis of a transaction and take customers as the direction. At the same time, it provides an idea that customers are value co-creators, emphasizing a two-way interaction between enterprises and customers.

As mentioned in Proposition 8, the service center must be customer-oriented. Keeson not only produces smart beds, but also incidental applications with detection and adjustment functions for consumers. Commodities become carriers of services and services become extensions of goods. While paying more attention to product efficacy, Keeson also establishes a two-way interaction with consumers to enable consumers to obtain a better user experience. On one hand, through self-service consumption, customers share physical data with professional doctors and put forward their needs for goods and services. Alternatively, enterprises actively improve their products and gradually realizes the customization of product design, sales, and services. On the other hand, enterprises collect confidential data from consumers with different characteristics, establish their own databases, and then build a healthy and professional Internet of Things technology enterprise to achieve high-level transformation from manufacturing to services. Similarly, as stated in propositions 6 and 7, although customers cannot transmit value, they can be value creators and cooperative producers of resources. In the future, consumers are more likely to promote business service innovation through value propositions. Breaking down the boundary between enterprises and consumers undoubtedly further stimulates Keeson's development.

### Smiling Curve and Value Chain

The smiling curve is a corporate competition strategy proposed by the founder of Taiwan's Acer Group Shi Zhenrong in “Reinventing Acer: Creation, Growth, and Challenges” in 1992, which was later revised as the “Industrial smiling curve.” The smiling curve is divided into the following three segments: left, middle, and right. The left segment is the R&D section of technology and patents. The middle segment is the assembly process and manufacturing link. The right segment is the marketing, service, and brand links. The entire curve is in the shape of a smile symbol. After a long period of research, it is suggested that high added value is reflected in the left and right ends of the curve, and low added value is reflected in the middle of the curve. Only by continuously moving and positioning in areas with high added value, can companies continue to develop and operate sustainably. However, Keeson not only emphasizes innovation in R&D, but also injects intelligence into the production process. Keeson is gradually moving toward the stage of intelligent manufacturing and is increasing the added value of its products. This enterprise already has 245 major patents, 224 domestic patents, and 40+ domestic and foreign invention patents. In addition, this enterprise cooperates with sleep experts and builds core sleep algorithms to improve its services.

The smile curve represents the vertical added value, while the value chain extends horizontally. In terms of strategic importance, the activities of a specific enterprise can be broken into several parts as follows: production, sales, incoming logistics, delivery logistics, after-sales service, supporting activities, etc. The network structure of these activities constitutes the value chain. A value chain is a link chain that generates value from a product or service. It is divided into a vertical value chain, a horizontal value chain, and an internal value chain. The vertical value chain refers mainly to the external value activity of the enterprise, that is, the value created and received by suppliers, customers, and channels. The horizontal value chain is the activity that generates value for enterprises, such as design, production, marketing, and delivery. It has the important function of linking production, sales, and service. The internal value chain categorizes and analyzes all the production and operation activities of an enterprise, and identifies strategic and value-added activities of the enterprise. These chains can jointly enhance the enterprise's core competitiveness.

**Figure 1:**
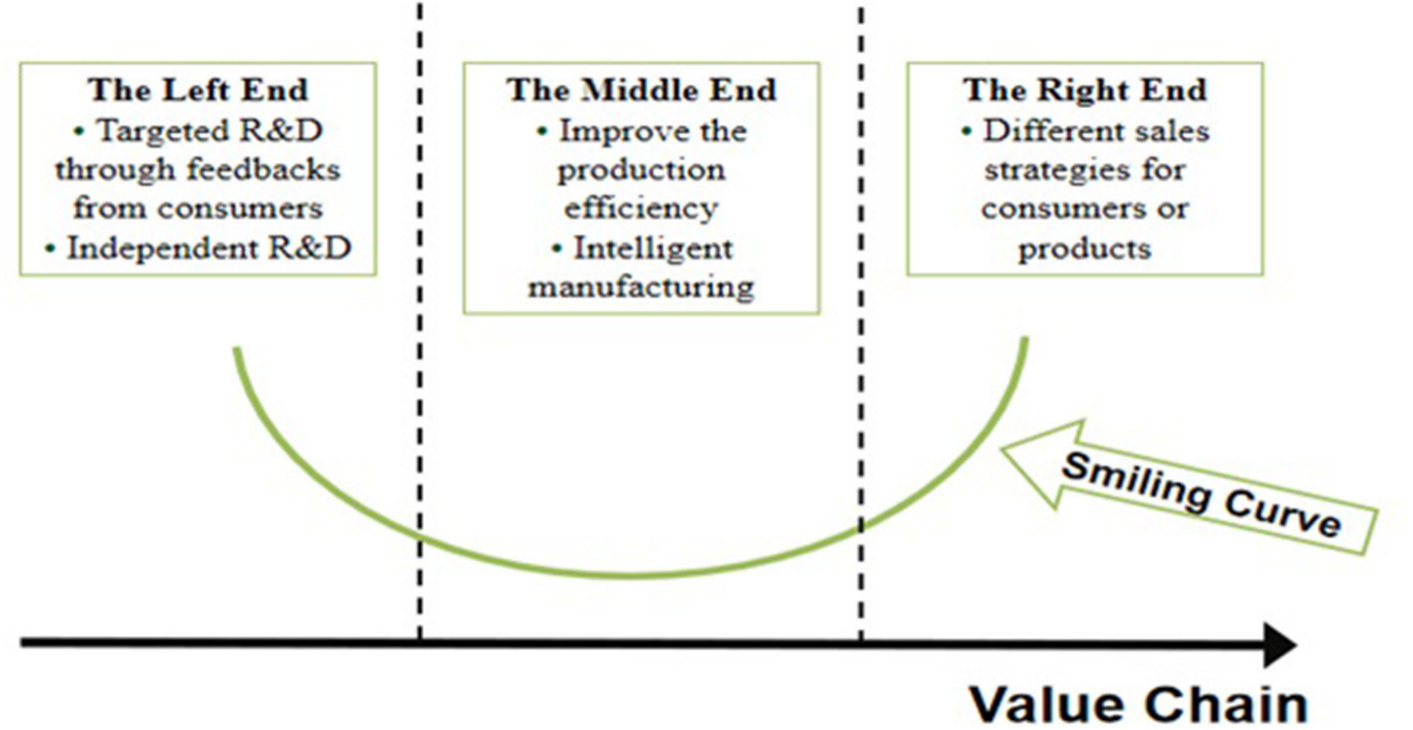
Six scenarios obtained by combining the corporate smiling curve with the value chain.

In this study, six scenarios were obtained to combine Keeson's development status (Figure 1). By opening up its smiling curve and the right end of the value chain, Keeson accepts more customer feedback and further connects the left end of the value chain, transforming from providing products to optimizing services, and brand building. Based on the mid-end intelligent manufacturing, Keeson takes a small step toward both ends of the value chain. While ensuring its own competitive advantages, it also integrates technology and services into the manufacturing industry to expand its brand influence. Whether in the left, middle, or right segments, companies have formed corresponding strategies to increase the added value of their products, thereby further improving their level on the smiling curve. This is a new idea of manufacturing servitization, and it is also one of the manifestations of boundless innovation in enterprises.

### Long Tail Theory

Chris Anderson first proposed the long tail theory in 2004. He points out that the future of business and culture lies not at the “head” of the traditional demand curve but at the infinite “tail.” The head of the demand curve is popular, while the demand distributed at the tail is personalized, scattered, and small. Although this segment was differentiated, small demand may seem like a long tail and does not dominate. However, the so-called long tail effect lies in its quantity. When all the non-popular markets are added up, a market larger than the popular market will be formed. Market data also show that not only does the general public have a demand for smart homes, but niche consumers are also looking forward to the production of targeted products.

The report generated by Keeson's questionnaire for consumers fully shows the functions demanded by consumers of different ages for mattresses. Some demands are for the relief of pain, others are for the adjustment of sleep habits, and still others are for specific design expectations. All of these needs reflect the subhabits of various consumer groups. The long tail effect emphasizes “individuation,” “customer power,” and “small profits and the big market.” In other words, there are small profits but a quick turnover. When the market is segmented into very small segments, the accumulation of these small segments will lead to a distinct long tail effect. Keeson achieves long-term stable development by occupying these sub-customary market segments and maintaining customers through continuous boundless information and service value transmission. At the same time, by segmenting markets, Keeson can focus on a specific target market or focus on a product and a service to create advantages.

### Springboard Theory

The springboard theory published by Luo and Tung illustrates the internationalization theory of emerging market enterprises based on strategic asset theory and ownership advantage (38). The springboard theory holds that internationalization is a springboard for improving the capabilities of multinational enterprises in developing countries. Through international expansion, multinational enterprises acquire the core technological assets of enterprises in developed countries through mergers and acquisitions (M&As) or purchases to eliminate the constraints of the home country system and market and avoid the disadvantages stemming from the lack of home country development. As a multinational enterprise in a developing country, Keeson knows that different countries and regions have different levels of acceptance regarding smart beds. From 2013 to 2018, Keeson focused on the US market, which accounts for more than 70% of its annual sales. Based on its sales experience in the USA, Keeson has expand its sales scope from 11 countries to 35 countries in 6 years.

The springboard theory indicates that there are asset-seeking and opportunity-seeking motivations behind the springboard behavior. The success of the springboard activity requires enterprises to adopt an integrated approach, to combine the resources obtained from the outside with the home country's basic advantages of emerging market companies. From a springboard perspective, transnational M&As are regarded as a vital means for latecomers to acquire strategic core assets. They often directly acquire advanced technology enterprises in developed countries and attach importance to the role of transnational M&As in acquiring tacit knowledge. Keeson first developed in the USA. Through the US market, it acquired resources that could not be obtained in its home market and absorbed many advantages of the US market. Keeson took this opportunity to raise the brand's popularity. Furthermore, Keeson uses the USA as a springboard to make up for its shortcomings, achieve technological catch-up, and narrow the gap with major international brands.

### Theoretical Integration

Now, we summarize the theoretical framework of this study. First, focusing on the long tail market is to provide personalized services to health and elderly care enterprises. It is not only related to the mainstream demand, but also related to secondary consumption habits. The long tail market is not the mainstream market, but it accounts for about 80% of the whole market. By collecting personalized services, the value transmission mechanism and service leading logic are introduced. Consumers not only get products and services, but also become service and product designers. At the same time, enterprises constantly update the database and improve products. This is a win–win scenario in which the value is transmitted in both directions.

In addition, we combine the smiling curve with the value chain. These two theories focus on value-added issues. This enterprise invested in R&D. With the discrepancy of the long tail market needs, the enterprise is perfecting technology and products. This intelligent manufacturing greatly improves productivity and technology. Regarding sales, on one hand, the value transmission mechanism adds a value premium to product design. On the other hand, this enterprise has cooperated with manufacturers, retailers, and the internet platform to accumulate customers and sales channels. All these intangible assets add value to products. Moreover, considering this enterprise's overseas expansion, the springboard theory is introduced.

Last but not least, all of the above theories are based on boundless innovation. With boundless thinking, the boundaries between services and products, producers and consumers are increasingly blurred. Sales/marketing, production, and R&D are closely connected with each other. Boundless application scenarios break the limits of time and space. Unlike traditional cognition, boundless innovation provides enterprises with unlimited development potential.

## Empirical Analysis

Next, we use a cluster analysis and a Bayesian model to select the target market and predict the expansion of overseas markets for Keeson. At present, the main target markets of Keeson are developed countries and regions, especially the USA. According to the export value of Keeson from 2013 to 2018, products exported to the USA over the years accounted for 92, 73, 75, 85, 82, and 88% of the total export value, with an average of approximately 80%.

Obviously, most of the products are exported to the US market. However, due to the existence of the trade issue between China and the USA, Keeson should expand its customer base as soon as possible, actively explore other overseas markets with relatively stable political and economic conditions, and avoid potential risks caused by the deterioration of the trading environment in a single market. Based on its superior position in the US market, Keeson can use the USA as a springboard for further expansion of its overseas market business and actively seek other potential markets. In recent years, combining a cluster analysis with Bayesian models has become a popular approach.

In the empirical analysis part, first, we classify the selected markets using a cluster analysis. Second, based on the cluster analysis, countries in the same category as the USA are selected as subjects of the Bayesian model. Then, a Bayesian model is applied to make probabilistic predictions regarding the relevant factors affecting the sales of smart beds. Finally, the target markets are classified again according to the results of the probabilistic prediction. At the same time, practical suggestions are provided for the expansion of overseas markets.

### Cluster Analysis Model

According to the sales data of Keeson in recent years, this enterprise expanded mainly in countries with relatively high levels of economic development. Keeson has a rich experience in trade environments, quality standards, market access conditions, and other aspects of economically developed countries. Therefore, it would be a better option to concentrate on expansion in markets with higher levels of economic development than to attempt to expand in less developed countries, which would take a lot of time and energy. This option is more efficient and has a more obvious effect. The number of smart beds sold in a country is inextricably linked to the country's level of economic development. Gross domestic product (GDP) is generally recognized as the best indicator of a country's economic development. Therefore, this study selects the top 30 countries in terms of GDP as the object of research.

First, the cluster analysis was performed in the top 30 countries. In this study, market development environment, market capacity, and market access barriers are the reference indicators, which are defined as first-level clustering indices (set as *X*1–*X*3). Specific subindices are defined as secondary clustering indices, which are set as *Xn*_1_-*Xn*__*i*_._ Then, to provide the basis for final clustering, the secondary indices under each primary index are clustered, analyzed, and assigned values according to the results. Finally, based on the assignment of the secondary indices, the model clusters the primary indices to obtain the final classification result (see Table 1).

**Table 1:** Selected indices for cluster analysis.

**Primary clustering indices**	**Clustering method of primary indices**	**Secondary clustering indices**	**Clustering method of secondary indices**
X1: Market development environment of adjustable beds	System clustering method based on the evaluation of secondary indices clustering results	• X11: Average annual GDP • X12: Average annual GDP growth rate • X13: Average annual merchandise trade as a percentage of GDP • X14: Average annual inflation rate	Systematic clustering method
X2: Market capacity of adjustable beds		• X21: Total population • X22: Average annual population growth rate • X23: Annual GDP per capita • X24: Annual GDP growth rate per capita • X25: Annual gross national income per capita	Systematic clustering method
X3: Market access barriers of adjustable beds		• X31: Legal rights strength index • X32:Corporate information disclosure index • X33: Ease of doing business index	Type 0 - 1 variable clustering method

#### A Cluster Analysis of the Secondary Indices

(1) A cluster analysis of the development environment of smart bed market

The market development environment is the basis for enterprises to decide whether to enter the country. The development environment of the smart bed market mainly depends on the level of local economic development. Therefore, the following four indicators are selected: average annual GDP, average annual GDP growth rate, average annual merchandise trade as a percentage of GDP, and average annual inflation rate. Considering that the data of a single year may affect the clustering effect due to fluctuations, the annual average index from 2009 to 2018 is selected.

According to the processing results of SPSS, countries can be divided into six categories based on the development environment of the adjustable bed market, where the USA, China, and Argentina make up one category; the Netherlands, Belgium, and the UAE make up one category; India, Turkey, Indonesia, Nigeria make up one category; and the remaining countries make up one category. According to the economic development degree of each country, we make a preliminary judgment and assign values. The USA is assigned 1; China is 2; the rest of the countries are 3; India, Turkey, Indonesia, and Nigeria are 4; the Netherlands, Belgium, UAE are 5; and Argentina is 6. Although the order of assignment can reflect to a certain extent the degree of national economic development, the main role of the assignment is to provide a classification basis for first-level cluster analysis.

(2) A cluster analysis of the market capacity of smart beds

To measure the market capacity of smart beds in a country, on one hand, it is necessary to consider the total population and growth rate of the country. On the other hand, we must fully consider the economic development situation of the country. Therefore, this study selects the following five indices: total population, average annual population growth rate, annual GDP per capita, annual GDP growth rate per capita, and annual gross national income per capita. Considering that the population index of a single year may affect the clustering effect due to fluctuations in specific years, this study uses the latest population data of 2018. The rest of the indices are selected from the average annual indices from 2009 to 2018.

According to the processing results of SPSS, countries can be divided into six categories based on the smart bed market capacity, China and India make up one category; Norway and Switzerland make up one category; Nigeria make up one category; Saudi Arabia and the UAE make up one category; Turkey, Indonesia, the Republic of Poland, Thailand, South Korea, Mexico, Iran, Argentina, Brazil, and Russia make up one category; and the remaining countries make up one category. Based on the economic development level and population of these countries, we can assign values to these six types of markets. The remaining countries are 1; China and India are 2; Turkey, Indonesia, the Republic of Poland, Thailand, South Korea, Mexico, Iran, Argentina, Brazil, and Russia are 3; Saudi Arabia and the UAE are 4; Norway and Switzerland are 5; and Nigeria is 6.

(3) A cluster analysis of the market access barriers of smart beds

For the clster analysis of barriers to access into the smart bed market, this study selects the following three indicators: the strength of legal rights index, the corporate information disclosure degree index, and the ease of doing business index. These data come from the World Bank. The legal rights strength index measures the extent to which collateral and bankruptcy laws promote lending activity by protecting the rights of borrowers and lenders. The index ranges from 0 to 12. Higher values indicate that collateral and bankruptcy laws are more conducive to obtaining credit. The corporate information disclosure index is the degree of investors protection based on the disclosure of ownership status and financial information. The index is ranked from 0 to 10. A greater value represents a higher degree of disclosure. The business convenience index ranks economies from 1 to 190, with the first place being the best. A higher ranking indicates that the regulatory environment is more conducive to doing business.

All variables in China are “1,” indicating low barriers to market access. According to the software processing results, China, Japan, France, Italy, South Korea, Spain, Indonesia, Saudi Arabia, Argentina, Norway, UAE, and Iran can be grouped into one category with a value of 1; Germany, Brazil, the Netherlands, Switzerland, and Austria make up a category with a value of 3. These countries have high barriers to entry. The rest of the countries are basically at the same level. They are classified in a category with a value of 2.

#### A Cluster Analysis of Primary Indices

According to the results of the secondary index analysis (see Table 2), this paper clusters on the primary index to determine the final classification.

**Table 2:** Selected indices for cluster analysis.

**Serial number**	**Countries**	**X1**	**X2**	**X3**
1	The United States	1	1	2
2	China	2	2	1
3	Japan	3	1	1
4	Germany	3	1	3
5	The United Kingdom	3	1	2
6	France	3	1	1
7	India	4	2	2
8	Italy	3	1	1
9	Brazil	3	3	3
10	Canada	3	1	2
11	Russia	3	3	2
12	Korea	3	3	1
13	Spain	3	1	1
14	Australia	3	1	2
15	Mexico	3	3	2
16	Indonesia	4	3	1
17	Netherlands	5	1	3
18	Saudi Arabia	3	4	1
19	Turkey	4	3	2
20	Switzerland	3	5	3
21	Poland	3	3	2
22	Sweden	3	1	2
23	Belgium	5	1	2
24	Thailand	3	3	2
25	Argentina	6	3	1
26	Austria	3	1	3
27	Norway	3	5	1
28	UAE	5	4	1
29	Iran	3	3	1
30	Nigeria	4	6	2

**Figure 2:**
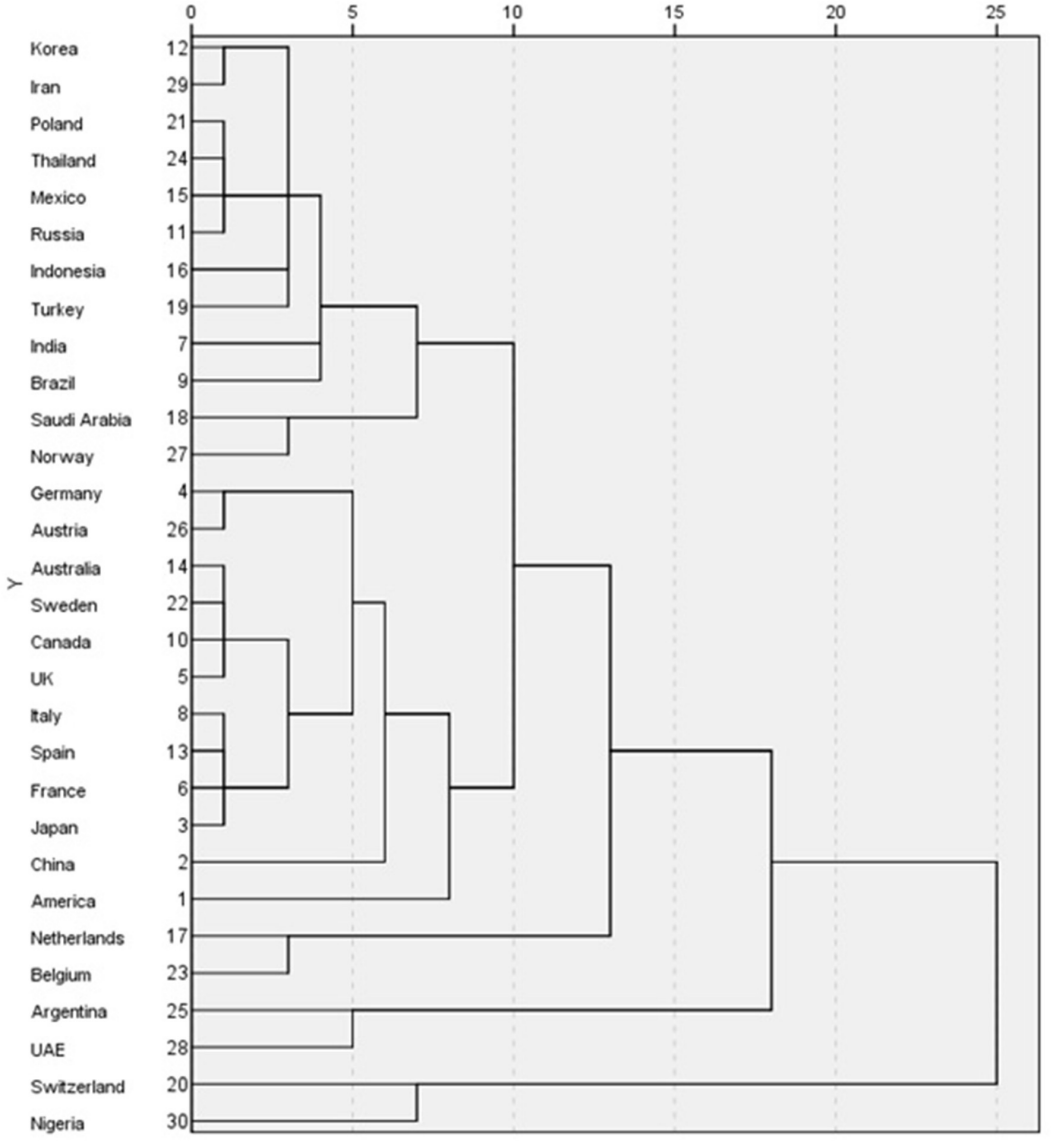
Tree diagram of smart beds market group.

The market for smart beds can be finally divided into the following five categories by SPSS processing: Switzerland and Nigeria make up one category; Argentina and UAE make up one category; Belgium and the Netherlands make up one category; the USA, China, Japan, France, Spain, Italy, the UK, Canada, Sweden, Australia, Austria, and Germany make up one category, and the rest of the countries are grouped into one category (see Figure 2). As Keeson developed the best in the US market, this study uses the USA as a springboard to select subjects. Finally, 12 countries that have the same clustering results as the USA are selected as research subjects.

### Bayesian Model

#### Bayesian Model Introduction

The Bayesian formula calculates the posterior probability based on the conditional probability formula and the total probability formula. If event *B* can only occur with one of the events, which are mutually incompatible with each other, that is, $B=\overset{n}{\underset{i=1}{\sum }}BA_{i}$, under the condition that event *B* must occur, the probability of the occurrence of event *A*_*i*_ can be expressed by the probability of event *A*_*i*_ and the conditional probability of the occurrence of event *B* under the condition of event *A*_*i*_, as follows:


(1) $$P(A_{i}|B)=\frac{P(A_{i})*P(B|A_{i})}{\sum_{i=1}^{n}P(A_{i})P(B|A_{i})}$$

To introduce the Bayesian model, this study transforms the event form (as shown in Formula 1) into a random variable form. A random variable θ is introduced, and it values are θ_1_, θ_2_, θ_3_, …θ_*k*_, where θ_*j*_ = θ(*A*_*i*_), namely, when *A*_*i*_ occurs, θ is θ_1_. If θ is divergent and has a prior distribution, π(θ_*j*_) is as follows:


(2) $$\begin{array}{r} \pi \left(\theta_{j}\right)=P\left(\theta_{j}\right)=P\left(A_{j}\right),\;j=1,2,3,\ldots ,k. \end{array}$$

Event is a random event. A random variable is defined: (as shown in Formula 1) and can be described as follows:


(3) $$\begin{array}{r} P\left(X|\theta_{j}\right)=P\left(X|\theta =\theta_{j}\right),\;j=1,2,3,\ldots ,k. \end{array}$$

It represents a kind of sample distribution. The sample size is *n* = 1. This sample distribution relies on a parameter θ, and parameter θ is a random variable. The sample distribution is recorded as *P*(*X*∣0). The random variable (Formula 4) can be deducted in the above way.


(4) $$\begin{array}{c} \begin{array}{c} P(\theta_{j}|X)=\frac{P(X|\theta_{j})\pi (\theta_{j})}{\sum_{j=1}^{k}P(X|\theta_{j})\pi (\theta_{j})},\;i=1,2,3,\ldots ,k. \end{array} \end{array}$$

The Bayesian formula can be used to solve a practical problem. Assuming that event B can occur under various conditions, assumptions about the nature of these conditions can be made as follows: These are hypothetical probabilities before the test (known as pretest probabilities), and we also know the probability of event B occurring under condition, i.e., (called conditional probabilities). By using the new information, the calculation result of the Bayesian formula finally modifies the pretest probability. This case makes a probabilistic prediction regarding the relationship between the average sleep time, average working time, population aging level, and the smart bed sales of the enterprise in each target country.

#### Application of the Bayesian Model

With a prior distribution to derive the posterior distribution, Bayesian estimation is a robust estimation method that can use small sample data to solve heterogeneity problems. The prediction result of the Bayesian model is better than that of ordinary regression models and is favored by scholars at home and abroad. Therefore, it is widely used in social science research (39). Some scholars are good at analyzing the influencing factors of economic phenomena, such as long-term national economic growth (40), real estate price fluctuation, and inflation inertia, with Bayesian models.

To reflect the sleep habits of people in the target country, this study selects three indicators. The three indicators are average annual working hours, average daily sleep time, and the degree of population aging in each country (see Table 3). Among the 12 subjects selected by clustering, the enterprise has not yet developed markets in Sweden and Austria. In addition, the purpose of this study is to expand overseas markets, and China, Sweden, and Austria are excluded. Therefore, the study includes only the remaining nine countries in the Bayesian model.

**Table 3:** Indices and data related to sleep habits.

**Serial number**	**Countries**	**Average working hours (hours)/year**	**Average sleep time (hours)/day**	**Degree of population aging (65 +)%**
1	The United States	1,790	7.16	9.68
2	Japan	1,719	6.35	26.02
3	Germany	1,371	7.175	21.12
4	The United Kingdom	1,674	7.26	18.12
5	France	1,482	7.335	18.94
6	Italy	1,725	7.125	22.36
7	Canada	1,706	7.295	16.15
8	Spain	1,691	7.125	18.88
9	Australia	1,665	7.235	14.98

(1) Forecast of the relationship between annual average working hours and smart bed sales

Average annual working hours in a country reflect the intensity of work in the country. Will labor intensity correlate with the sales of smart beds? The sales data in this case were obtained through a field survey of Keeson.

The processed data in Table 4 are calculated using the Bayesian formula. Under the condition that the average working hours are different in different stages, the probability of occurrence in countries with a high smart bed sales value (the annual sales value exceeds ¥10 million) is shown in Table 5.

**Table 4:** Analysis of average working hours and sales of smart beds.

**Average working**		**Count**	**Sales grade**		
**hours (hours)/year**			**(Unit: 10,000 RMB)**		
			**High (>1,000)**	**(Medium, 10–1000)**	**Low (<10)**
A1	>1,700	4	2	1	1
A2	1,600–1,700	3	1	0	2
A3	<1,600	2	0	1	1
	Total	9	3	2	4

**Table 5:** Probability of countries with high smart bed sales in different working time periods.

**Average working hours (hours)/year**	**Probability (high-volume countries)**
>1,700	0.67
1,600–1,700	0.33
<1,600	0

According to the results, the longer the average annual working hours in a country, the higher the sales of smart beds in that country. On one hand, long working hours reflect a relatively higher income level and stronger purchasing power for smart beds. On the other hand, due to long working hours, people generally hope to get a high-quality sleep at night, so the demand for smart beds is greater. In summary, annual average working hours and the sales of smart beds show a positive correlation. Therefore, when Keeson further explores new markets, it should take the average annual working time of the target country into consideration and try to select countries with longer average annual working hours.

(2) Forecast of the relationship between the average daily sleep time and the sales of smart beds

Sleep time refers to the time required for a person's natural physiological needs. During this period, physical strength is restored, eyes are closed, and the cerebral cortex is at rest. Sleep can be divided into intermittent sleep and continuous sleep. Sleep time refers to the total amount of time spent asleep in a day, that is, the sum of all sleep times. Is there a certain relationship between the average daily sleep time and the sales of smart beds? This study calculated the probability with a Bayesian formula.

The processed data in Table 6 are calculated using the Bayesian formula. Under the condition that the average sleep time is different in different stages, the probability of occurrence in countries with a high smart bed sales value (the annual sales value exceeds ¥10 million) is shown in Table 7.

**Table 6:** Analysis of average sleep hours daily and sales of smart beds.

**Average sleep**		**Count**	**Sales grade**		
**time (hours)/day**			**(Unit: 10,000 RMB)**		
		**High (>10,00)**	**(Medium10–1000)**	**Low (<10)**
A1	>7.2	4	2	1	1
A2	7.1–7.2	4	1	0	3
A3	<7.1	1	0	1	0
	Total	9	3	2	4

**Table 7:** Probability of countries with high smart bed sales in different sleep time periods.

**Average sleep time (hours)/day**	**Probability (high-volume countries)**
>7.2	0.67
7.1–7.2	0.33
<7.1	0

The probabilistic results show that the longer the average sleep time is, the higher the probability of countries with high sales of smart beds being in this interval. This phenomenon can be attributed to the fact that people in countries with longer average sleep times are inclined to pay more attention to sleep and then have higher requirements for sleep quality, eventually increasing the demand for smart beds. Therefore, when Keeson further develops the market, it should also take the average daily sleep time of the target country as a factor to consider, and try to select countries with longer average sleep times.

(3) Forecast of the relationship between population aging and smart bed sales

Population aging refers to the trend that the proportion of the elderly population increases correspondingly due to the decrease in the young population and the increase in the elderly population in the total population caused by the decrease in the fertility rate and the extension of life expectancy. As product users are mainly the elderly, will the corresponding increase in the proportion of the elderly in the population promote the increase in smart bed sales?

The processed data in Table 8 are calculated using the Bayesian formula. Under the conditions of population aging being different in different stages, the probability of occurrence in countries with a high smart bed sales value (the annual sales value exceeds ¥10 million) is shown in Table 9.

**Table 8:** Data analysis of degree of population aging and sales of smart beds.

**Degree of**	**Count**	**Sales grade**		
**population aging**		**(unit: 10,000 RMB)**		
		**High (>1,000)**	**Medium (10–000)**	**Low (<10)**
A1	>25%	4	0	2	2
A2	20–25%	5	3	0	2
A3	<20%	0	0	0	0
	Total	9	3	2	4

**Table 9:** Probability of countries with high adjustable bed sales in population aging degree ranges.

**Degree of population aging**	**Probability (high-volume countries)**
**>25%**	**0**
**20–25%**	**1**
** <20%**	**0**

The probabilistic results show that there is no significant correlation between population aging and smart bed sales. Therefore, when Keeson expands and selects target countries, the population aging index has little effect on the selection and has no reference significance.

To sum up the three indices, when Keeson explores overseas markets and selects target countries, it should fully consider the average sleep time and average work time of each country. The longer the average working time and the average sleeping time in a country, the more favorable the market in that country is for smart bed sales.

### Forecast of Market Classification Results

According to the predicted result, it can be concluded that average sleep time and average working time have a certain influence on smart bed sales. We can divide the target markets into three categories based on these indicators (see Table 10). The cutoff point for average working time is 1,690 h/year, and the cutoff point of average sleeping time is 7.16 h/day.

**Table 10:** Target market classification based on Bayesian Model.

**Categories**	**Features**	**Countries**
The first category	• Long average worksing time • Long average sleep time	The United States, Canada
The second category	• Long average working time • Short average sleep time	Japan, Italy, Spain
The third category	• Short average working time • Long average sleep time	UK, Australia, France, Germany

The first type of market is the market that the enterprise needs to focus on first. It has absolute advantages in terms of the market environment, market potential, ease of entry, and consumer characteristics. Although the advantages of the second and third markets are not obvious compared to the first market, they have certain advantages over other countries that are not in the same category as the USA. In the second and third markets, although there is not much difference in the average sleep time in each country, the average number of working hours in the second market is obviously higher than that in the third market. According to the Bayesian model, the average number of working hours has a positive correlation with the sales of smart beds. Therefore, the second type of market should be prioritized over the third type of market.

## Value Mining Strategy for the Global Big Health Market

### Development Strategies of the First Market

Based on the results of Bayesian analysis, the USA and Canada are listed as the first market due to their higher labor intensity and longer sleep time. These markets accounted for 90% of Keeson's total sales in 2018. The US and Canadian markets are high-volume and high-growth markets, which are suitable for the priority development strategy—giving priority to the resources required to expand economic scale and increase market share.

#### Using the USA as a Springboard

The existing products of Keeson have certain advantages in the USA. From 2013 to 2018, sales in the USA accounted for more than 70% of Keeson's total sales. In addition, the USA is the most important consumer market for smart beds in the world. According to a survey conducted by McKinsey, the size of the sleep health industry in the USA has reached $30–40 billion, with an annual growth rate of 8%. Therefore, it is reasonable to choose the USA as a springboard.

Keeson has emphasized the acquisition of tacit knowledge from the USA. At present, as a good starting point, Keeson has established long-term cooperation with enterprises such as SSB, TSI, and Costco. SSB and TSI are the two giants of the US mattress industry with huge market shares and important end-customer channels. Keeson should regularly summarize customer preferences, quality feedback, institutional, and cultural differences between countries, which can provide the information of the end-customer demand and internalize the advantages of its partners, especially in terms of technology, brand, talent, operation, and marketing channels.

Keeson should choose markets that are similar to the USA as potential target markets. Through the abovementioned cluster analysis, 12 countries are selected, including the USA, China, Japan, France, Spain, Italy, the UK, Canada, Sweden, Australia, Austria, and Germany. These countries are similar to the USA in terms of market environment, market capacity, and entry barriers. With the experience accumulated in the USA, Keeson can focus on promoting products in these 12 countries to increase the possibility of success.

#### Establishing an Innovation Mechanism

The smart bed industry in the first category is converging and highly competitive. Only continuous learning and innovation can strengthen a company's foothold. The funds and scale of local leading companies in the USA have reached a considerable level. The power structure of the value chain is basically settled, forming a situation in which several mattress brands monopolize the market. Therefore, Keeson needs to improve its product R&D, design, production, sales, service, and other aspects to establish its own advantages. Keeson should establish a systematic innovation mechanism using the information obtained.

To a great extent, the efficiency of capturing and transforming external knowledge depends on the innovation culture of enterprises. As of 2018, Keeson's products have been sold in 36 countries around the world. These markets should not be viewed simply as a way to gain cash flow, but as a way to learn about the company's clients. At Keeson, an incentive system for talent should be established. For example, first, an innovation institute should be established and industry experts should be regularly invited to interpret the latest industry trends. Second, Keeson should regularly collect customer feedback in the department of after-sales service. At the same time, a fund should be set up to reward talents for patent applications, breakthroughs in major projects, and other special contributions.

### Development Strategies of the Second Market

The second market is Japan, Italy, and Spain, where the market concentration of smart beds is relatively low, and enterprises can seize the market by promoting their own strengths. For example, Japan is known for its longer working hours and higher social pressure. According to the Guardian, Japan suffers 138 billion in economic losses each year due to insomnia, and the sleep economy is on the rise. However, compared to the USA, there is little gap between the brands because Japan's smart beds market started later. Therefore, Keeson can start from the right end of the smiling curve, occupy an active position on the sales side, and gradually establish its own brand.

#### Boundless Relationship Between Enterprises and Consumers

In traditional service value theory, consumers are not involved in value creation. Based on big data, Keeson should gradually introduce customer feedback into the industry chain to achieve a two-way interaction between the enterprise and consumers. The enterprise processes the massive data gathered, extracts the key information using big data analysis technology, and discovers the characteristics of products preferred by different consumer groups.

For example, according to the survey conducted by Keeson on consumers, consumers aged 18–29 prefer low-priced, lightweight mattresses with environmental protection. However, consumers aged 30–39 pay more attention to the service quality of product delivery and installation. Machine learning algorithms can be used to provide accurate feedback on the needs of consumers to the enterprise in a timely manner. In the process of information transmission, consumers not only consume goods and services but also become co-creators of value. Different user demands will force product improvement at the supply end, providing endless power for a product innovation from Keeson.

#### Boundless Scene Marketing

Keeson should choose scenes in which smart beds are the starting point for marketing, including hotels, sleep research institutions, medical facilities, nursing rooms, nursing homes, etc. Scene marketing involves not only selling products but also a new lifestyle, and the establishment of this lifestyle is entirely based on the characteristics of the consumers.

For example, in sleep research institutions, smart beds can be used as a channel to obtain sleep data. In medical institutions, sleep data can be used to provide evidence from patients to doctors to assess their current physical conditions. In the field of old-age care, smart beds can be used to establish personal health information files. In young people's social circles, sleep can appear alongside fitness, travel, and baking as a testament to healthy living. Although differentiated demand appears as a long tail on the demand curve, according to the long tail effect, the combination of all non-popular markets form a considerable market.

### Development Strategies of the Third Market

The third type of sales market is England, Australia, France, and Germany. Australia is an important country in Oceania, while Britain, France, and Germany are located in Europe. Based on the favorable income levels and consumption concepts, the demand for smart beds in these countries is large. Moreover, in this market type, a few brands do not monopolize the whole market. Therefore, Keeson could pull ahead of competitors in terms of product R&D and market positioning.

## Implications and Suggestions

### Implications From Keeson

Servitization and intelligence are both boundless innovation paths for Keeson and serve as good examples. Keeson's achievements rely on in-depth thinking regarding boundless innovation. This thinking not only considers product innovation and upgrades, but also consumer value. Keeson's products have become service carriers, which are also part of the line extension. While paying more attention to product functions, Keeson also establishes a two-way interaction with consumers who can obtain a better experience. The success of Keeson lies in breaking down the boundary between enterprises and consumers and allowing consumers to create value together.

The intelligentization of Keeson shows that new application scenarios require new boundless innovation thinking strategies. Under the trend of digitalization and intelligence, intelligent manufacturing has brought new application scenarios to traditional manufacturing enterprises. The rapid development of information and communication technology is changing people's lifestyle and work style, and is changing the way enterprises operate. In the era of big data, the manufacturing industry should actively embrace the intelligence trend, use boundless innovative thinking to promote the intelligent upgrading of products and services, and further promote the development of intelligent manufacturing in the industry.

### Suggestions for Health and Elderly Care Enterprises

Health and elderly care enterprises need to formulate boundless innovation strategies suitable to their own development strategies and corporate goals. Recently, the COVID-19 epidemic has spread rapidly around the world, placing pressure on the growth of the global economy and trade. In the short term, these unfavorable factors will have a certain negative impact on the overseas business of health and elderly care enterprises. However, in the long run, after suffering the effects of the epidemic, consumers will pay more attention to health and smart home furniture. Due to the increase in time spent at home, products such as smart beds that can improve the home environment and enhance the quality of life of consumers are more likely to be favored by consumers.

During the epidemic, on one hand, health and elderly care enterprises can adopt a service without face-to-face contact with users. Consumers can specify service hours and locations by phone, app, and other methods. At the same time, health and elderly care enterprises can take measures to solve the problems of distribution and maintenance for the consumer isolated at home, such as recording the whole service process, disinfecting the goods layer by layer, and holding health certificates for employees. The boundless innovation of health and elderly care enterprises should not only aim at product manufacturing, but also consider the service value transmission method.

### Limitations and Future Research

First, the limitation of this paper is that this research focuses on China's leading health and elderly care enterprises. This research has certain typicality, but it also has certain limitations. Second, this paper adopts the case analysis method to deeply analyze the successful practices of typical enterprises. The universality of the research results is comparatively weaker, but in view of the unavailability of primary data of many enterprises, this paper still has reference significance for enterprises' overseas market value mining mode.

In future research, it is necessary to combine various research methods to comprehensively explore the overseas market value mining mode and mechanism of health and elderly care enterprises, and put forward more effective suggestions.

## Data Availability Statement

The original contributions presented in the study are included in the article/supplementary material, further inquiries can be directed to the corresponding author/s.

## Author Contributions

JG, RG, and YunW contributed to the conception of the manuscript and wrote this manuscript. YM and KY collected the materials and data. HX and YuW contributed to data analysis or interpretation. All authors contributed to this paper and approved the submitted version.

## Funding

This work was supported by Jilin Provincial Social Science Fund (2022J15).

## Conflict of Interest

The authors declare that the research was conducted in the absence of any commercial or financial relationships that could be construed as a potential conflict of interest.

## Publisher's Note

All claims expressed in this article are solely those of the authors and do not necessarily represent those of their affiliated organizations, or those of the publisher, the editors and the reviewers. Any product that may be evaluated in this article, or claim that may be made by its manufacturer, is not guaranteed or endorsed by the publisher.
